# A Status Review on Health-Promoting Properties and Global Regulation of Essential Oils

**DOI:** 10.3390/molecules28041809

**Published:** 2023-02-14

**Authors:** Tareq M. Osaili, Dinesh Kumar Dhanasekaran, Falak Zeb, MoezAlIslam E. Faris, Farah Naja, Hadia Radwan, Leila Cheikh Ismail, Hayder Hasan, Mona Hashim, Reyad Shaker Obaid

**Affiliations:** 1 Department of Clinical Nutrition and Dietetics, College of Health Sciences, University of Sharjah, Sharjah P.O. Box 27272, United Arab Emirates; 2 Sharjah Institute for Medical Research, University of Sharjah, Sharjah P.O. Box 27272, United Arab Emirates; 3 Department of Nutrition and Food Technology, Faculty of Agriculture, Jordan University of Science and Technology, P.O. Box 3030, Irbid 22110, Jordan

**Keywords:** essential oils, anti-inflammatory, anti-cancer, metabolic health, obesity

## Abstract

Since ancient times, essential oils (EOs) have been known for their therapeutic potential against many health issues. Recent studies suggest that EOs may contribute to the regulation and modulation of various biomarkers and cellular pathways responsible for metabolic health as well as the development of many diseases, including cancer, obesity, diabetes, cardiovascular diseases, and bacterial infections. During metabolic dysfunction and even infections, the immune system becomes compromised and releases pro-inflammatory cytokines that lead to serious health consequences. The bioactive compounds present in EOs (especially terpenoids and phenylpropanoids) with different chemical compositions from fruits, vegetables, and medicinal plants confer protection against these metabolic and infectious diseases through anti-inflammatory, antioxidant, anti-cancer, and anti-microbial properties. In this review, we have highlighted some targeted physiological and cellular actions through which EOs may exhibit anti-inflammatory, anti-cancer, and anti-microbial properties. In addition, it has been observed that EOs from specific plant sources may play a significant role in the prevention of obesity, diabetes, hypertension, dyslipidemia, microbial infections, and increasing breast milk production, along with improvements in heart, liver, and brain health. The current status of the bioactive activities of EOs and their therapeutic effects are covered in this review. However, with respect to the health benefits of EOs, it is very important to regulate the dose and usage of EOs to reduce their adverse health effects. Therefore, we specified that some countries have their own regulatory bodies while others follow WHO and FAO standards and legislation for the use of EOs.

## 1. Introduction

Essential oil (EO) origins are believed to be from ancient China and Egypt [1]. They are extracted from various plant parts, such as wood, leaf, bark, and stem [2]. The recent trend is also shifting towards the use of natural or plant-based therapeutic products [3]. Among these plant-based products, the usage of EOs is 70%, which is the highest compared to other plant-based therapeutics [4]. Up until now, more than 3000 EOs have been extracted, mostly from families including *Lamiaceae*, *Rutaceae*, *Myrtaceae*, *Zingiberaceae*, and *Asteraceae*. Among them, more than 300 EOs are commercialized in the fragrance and food markets, with anticipated growth reaching more than $15 billion by 2025 [5].

EOs have bioactive compounds, which endow them with various beneficial properties [6]. Recently, Eos and their derivatives have gained attention due to their good tolerability and effectiveness in the prevention and treatment of diseases, including cancer and the metabolic syndrome, in both animal and human studies [7]. For instance, lavender EO has a highly anti-cancer and anti-mutagenic effect on HepG2 and A549 human cell lines [8]. Moreover, these bioactive compounds have powerful anti-septic, anti-inflammatory, antibacterial, anti-oxidative, and immune-boosting properties [9]. A recent study revealed that EOs from thyme species have strong anti-oxidant and anti-microbial activities and showed action against multi-resistant bacteria known as *Acinetobacter baumannii* [10]. Another study demonstrated that EOs of citrus hystrix DC. peel have noticeable anti-inflammatory effects against human melanoma in skin cells [11]. It is believed that the anti-obesity and anti-inflammatory properties of EOs may reduce the burden of metabolic diseases [12]. EOs have also been used in edible food films and coatings on food packages due to their biodegradable nature and bioactive properties. They consist of volatile compounds that help improve the antioxidant and antimicrobial properties of the food [13,14].

On the other hand, some extent of toxicity could be attributed to the consumption of EOs. A recent study demonstrated that EOs from various plant sources disrupted the endocrine hormonal levels in pregnant women [15]. Therefore, the usage of EOs is regulated by various international and local bodies (discussed in the last part of this review). This review briefly discusses the antioxidant, antimicrobial, anti-inflammatory, and metabolic effects of EOs and the regulations regarding their utilization.

## 2. Extraction and Chemical Composition of EOs

EOs are a complex mixture of volatile and semi-volatile organic compounds that determine the plants’ aroma, fragrance, and flavor [16,17]. To obtain a pure EO, firstly, distillation is performed, after which the pure EO bioactive component is extracted from the distillate. Distillation could be performed via steam, water, organic solvents, or microwaves. Meanwhile, the extraction could be performed using supercritical carbon dioxide, high-pressure solvent, ultrasound, and photonic processes [18,19]. Steam distillation is widely used for the extraction of EOs on a commercial scale [20]. An impure distillate may consist of up to 20–100 different plant secondary compounds [21].

EOs are usually lipophilic (fat-soluble) in nature but can be water-soluble. This would depend on the polar components present in the oil. The bioactive compounds that give EOs their characteristic features belong mainly to two chemical classes: terpenoids and phenylpropanoids. Terpenoids (monoterpenoids and sesquiterpenoids) are composed of hydrocarbon molecules. The simplest terpenoid molecule is the monoterpene, which is composed of 10 carbon atoms with a hydroxyl group present at no specific location along the chain. Examples of monoterpenes extracted from different plants include geraniol, terpineol (lilacs), limonene (citrus fruits), myrcene (hops), linalool (lavender), or pinene (pine trees) [22]. Sesquiterpenoids are compounds consisting of 15 carbon atoms with the molecular formula C_15_H_24_. They are named after the Latin word “sesqui”, meaning one and a half. A common example is a ginger extract, which contains the sesquiterpenoid compound called zingiberene. On the other hand, phenylpropanoids/shikimates are abundant in certain plant species. The specific odor and flavor of the plants could be attributed to these compounds [23,24,25].

**Figure 1:**
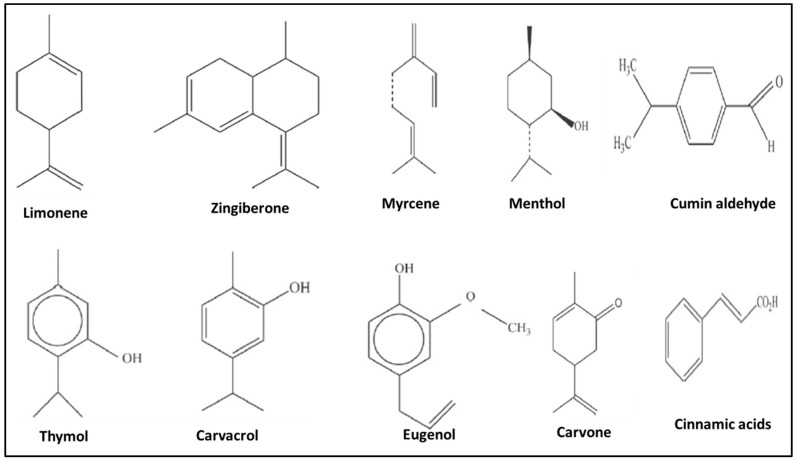
Molecular structures of some common essential oil compounds.

The other organic compounds inherent to EOs may be classified under functional groups such as alkenes (myrcene), alcohols (menthol), aldehydes (cumin aldehyde), phenols (thymol, carvacrol, and eugenol), ketones (carvone), and carboxylic acids (cinnamic acid) [17,24]. The structures of some common essential oil (EO) compounds with different functional groups are presented in Figure 1. The chemical/ bioactive compound composition of EOs differs among plant species and varies based on several factors such as geographical location, stage of plant maturity, season, geography, and part of the plant from which the EO has been extracted, amongst a myriad of other factors [26,27]. The bioactive compounds of EOs have been observed to undergo degradation and lose their pharmacological properties when exposed to factors like light, oxygen, or high temperatures [28].

## 3. Therapeutic Effects of EOs

### 3.1. Antioxidant, Anti-Inflammatory, and Anti-Cancer Activities of Essential Oils

Recent cumulative research suggests that EOs exhibit anti-inflammatory, antioxidant, and anticancer properties in cell and animal models [3,29,30]. Inflammation is a normal response to tissue damage caused by stimuli that could be biological, chemical, or physical [31]. EOs decrease the prerequisite compounds that exaggerate the inflammation process, namely, reactive oxygen species (ROS) and nitrogen species, NF-κB, and proinflammatory cytokines [32]. A comprehensive review of EOs revealed that oils extracted from lemon (Citrus Limon) fruit peels and leaves exerted antioxidant and radical scavenging activities under in vitro conditions [33]. Moreover, it was observed that the monoterpene hydrocarbon fraction was positively correlated with antioxidant activity [33]. Citrus EOs, namely, mandarin, wilking, and clementine, exhibited antioxidant activity by acting as free radical scavengers in a dose-dependent manner [34]. Edema is a pathological feature of inflammation [35]. Carrageenan, a pro-inflammatory agent, was used to induce paw edema in rats, while bergamot EO was applied for its anti-inflammatory effect. It was observed that the bergamot EO significantly decreased the inflammatory markers, namely, prostaglandin (PGE2) and nitrite/nitrate levels, as well as interleukin (IL)-1, IL-6, and tumor necrosis factor (TNF-α) [35]. On a similar note, sesame seed oil decreased lipid peroxidation and nitric oxide production besides increasing antioxidant enzymes such as superoxide dismutase (SOD), glutathione (GSH), GSH peroxidase (GPx), and catalase (CAT) by binding the fatty acids in sesame seed with COX-2 and PGE2 [36]. The COX-2 enzyme activates pain (a symptom of inflammation) mechanisms in the human body. The phytochemical components inherent to sesame seed oil, like sesamol, sesamin, and sesamolin, are responsible for their antioxidant and anti-inflammatory properties [37]. A clinical trial was conducted to investigate the effect of sesame oil on 40 hypertensive male and female patients aged 35–60 years [38]. The results demonstrated that sesame EO decreased lipid peroxidation and increased antioxidant activity [38]. Likewise, eugenol—a bioactive compound in clove EO—decreased rat paw swelling and synovial immune cell infiltration [39]. EOs of the oregano flowering plant (O. *vulgare*) have also been recognized for their antioxidant potential [40,41]. In contrast, it was also observed that bergamot EO increased intracellular ROS production induced by N-formyl-Met-Leu-Phe in the presence of extracellular Ca^2+^ chelators. Thereby, the pro-inflammatory potential of EOs also needs to be carefully considered for any future clinical applications [42].

Cancer may significantly increase the fatality rate of the disease by compromising the immune system. The abundant bioactive components in EO may offer immune-boosting activity in the treatment of cancer [43]. Navel orange EO exhibited good anti-cancer potential against lung cancer and prostate cancer [44]. Similarly, non-volatile fractions of bergamot EO, such as coumarins and furocoumarins, showed anti-mutagenic activity [45]. EOs from Croton flavens leaf were observed to be effective on human lung carcinoma and human colon adenocarcinoma cell lines, respectively [46]. Another study identified that an EO from *Curcumae rhizoma* (CR) inhibited the growth of colon cancer by improving tumor vessel structure, reducing angiogenesis in tumors, and normalizing tumor vessels in both in vitro and in vivo models [47]. Similarly, a recent study demonstrated that oregano EO-based nanoemulsion increased prostate cancer cell death through apoptosis and decreased lipid droplet accumulation via targeting HMGCR, FASN, and SREPB1 signals in vitro [48].

**Figure 2:**
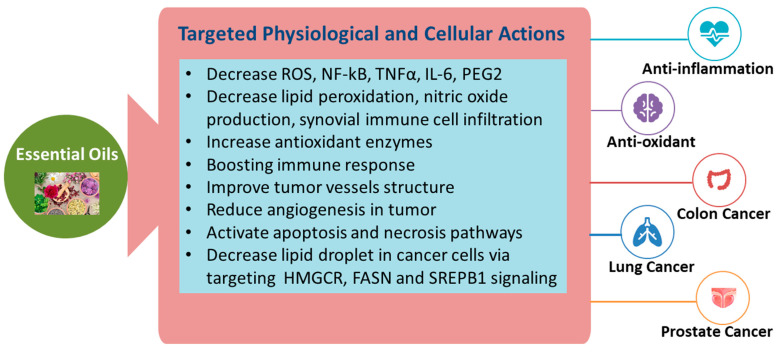
Targeted actions of EOs for the reduction of inflammation and cancer.

The ability of the EOs to exhibit these bioactive properties could be attributed to their composition of terpenes, hydrocarbons, alcohols, aldehydes, ketones, and esters [49,50,51]. Usually, the method of extraction used to obtain the EO is determined according to the purpose of use. Due to the lipophilic nature of EOs and their low molecular weight, they can easily cross cell membranes or even alter the membrane composition and increase/decrease membrane fluidity [52]. Consequently, changes within the membrane structure lead to the leakage of ions and cytoplasmic molecules, reduced ATP production, and loss of mitochondrial potential, eventually resulting in cell death [53]. Moreover, EOs, upon entering the cell membrane, activate apoptosis and necrosis pathways, resulting in cell death, cell cycle arrest, and loss of function of essential organelles [53]. EOs encourage the condensation and fragmentation of nuclei and chromatin, causing a loss of cristae in the mitochondria, which eventually leads to cell apoptosis [54]. In conclusion, EOs may reduce inflammation, oxidative stress, and cancer development through targeted actions both at the physiological and cellular levels, which are summarized in Figure 2.

### 3.2. EOs: A Remedy for the Management of Metabolic Syndrome

Metabolic syndrome (MetS) is a group of risk factors that predispose an individual to cardiovascular disease and type II diabetes mellitus of metabolic origin. Typical MetS risk factors include obesity, high blood pressure, high blood sugar, and dyslipidemia (high triglycerides and/or low high-density lipoprotein cholesterol) [55]. The pathophysiology and the exact etiology of the development of MetS are not very clear [56]. However, MetS has been associated with increased oxidative stress [57] and is characterized by elevated inflammatory markers [58]. Furthermore, obesity and insulin resistance lead to an increase in proinflammatory cytokines such as TNF-α and IL-6.

#### 3.2.1. EOs and Obesity

Obesity could be classified as the primary initiating factor of MetS, and attaining a healthy body weight is one of the first lines of treatment. Multiple plant-derived EOs, which are rich sources of volatile organic compounds, have long been used for the complementary treatment of obesity [59]. Several in vitro and in vivo studies suggest that the anti-obesity effects of EOs are achieved by the down-regulation of adipogenic transcription factors such as PPARγ and CEBPα at both protein and mRNA levels, the elevation of the plasma glycerol concentration (a marker of lipolysis), as well as the suppression of fat accumulation and intracellular triglycerides [60]. The EOs from the Citrus family have been widely investigated for their anti-obesity properties [60]. In an animal study, sweet orange EO was suggested as a potential dietary supplement for weight loss, since it decreased the expression of peroxisome proliferators-activated receptor-γ (PPARγ), upregulated uncoupling protein 2 (UCP-2), hormone-sensitive lipase (HSL) and carnitine palmitoyltransferase I (CPT-I), and inhibited the expression of acetyl-CoA carboxylase (AAC) [61]. The bioactive compound (+)-limonene, extracted from citrus peel EO, exhibited anti-obesity properties [62]. Moreover, grapefruit and citrus EOs are commonly used in aromatherapy for weight loss programs [63,64].

#### 3.2.2. EOs and Diabetes

Diabetes is a common complication of MetS. It is important to note that EOs may help in the management of diabetes mellitus (DM), but they cannot be used as a cure. The EOs exhibit their antidiabetic action by inhibiting α-amylase and α-glucosidase enzymes, and by increasing insulin sensitivity or/and insulin secretion [65]. EOs can also be used to reduce common complications of diabetes, such as ulcers and loss of skin integrity, and can decrease the duration of the infection [66]. EOs from Asian ginseng (*Panax quinquefolius*), fenugreek (*Trigonella foenum-graecum*), and aloe (*Aloe vera*) have been observed to improve glucose tolerance [67]. Lavender EO (*Lavandula stoechas* L.) was protective against diabetes and decreased the associated renal and hepatic injuries in diabetic rats [68]. A mixture of cinnamon bark (*Cinnamomum zeylanicum*), cumin (*Cuminum cyminum*), fenugreek (*Trigonella foenum-graecum*), and oregano (*Origanum vulgare*) was observed to lower glucose levels by enhancing insulin sensitivity [69]. One animal study suggested that bioactive compounds in cinnamon (*Cinnamomum veru*) can regulate adipocyte gene expression to improve glucose transport and insulin signaling [70]. Under in vitro conditions, hydro-distilled EO from clove buds inhibited the activities of α-amylase and α -glucosidase [71]. However, a meta-analysis identified only one randomized controlled study on the impact of EO on diabetes and thereby, a conclusive statement that provides evidence-based health claims on this issue cannot be made [72].

#### 3.2.3. EOs and Hypertension

Hypertension is another hallmark risk factor for the development of MetS. Antihypertensive medications and lifestyle modifications are the primary treatments for hypertension [73]. Preliminary studies indicate that EOs are effective in decreasing blood pressure and heart rate [74,75]. Ref. [74] blended four EOs, namely lavender (*Lavandula officinalis*), ylang-ylang (*Cananga odorata*), marjoram (*Origanum majorana*), and neroli (*Citrus aurantium*). The immediate and long-term effects of the EOs were evaluated by monitoring the 24-h ambulatory blood pressure and salivary cortisol levels, respectively. It was observed that the EO blend significantly decreased the daytime blood pressure and salivary cortisol concentration. However, there was an insignificant decrease in nighttime blood pressure [74]. Carvacrol, the major component of oregano EO, may help decrease blood pressure as it causes peripheral vasodilatation and inhibits the contraction elicited by intracellular Ca^2+^ influx through CAV (voltage-dependent calcium) channels and transient receptor potential (TRP) channels [76,77,78,79]. A systematic review indicated no significant effect of inhaled EOs on blood pressure reduction in patients with hypertension [80].

#### 3.2.4. EO and Dyslipidemia

Dyslipidemia is one of the major contributors to MetS. Lemon balm EO (*Melissa officinalis*) decreased triglycerides and reduced the expression of genes involved in fatty acid synthesis [74]. The purple yam (*Dioscorea alata* L.) was reported to be effective in controlling adipose tissue mass and increasing high-density lipoprotein cholesterol (HDL-C), decreasing triglyceride (TG), total cholesterol (TC), and low-density lipoprotein cholesterol (LDL-C) concentrations associated with gut microbiota modulation [81]. The antihyperlipidemic effect of EOs could be due to their ability to activate lipoprotein lipase [65].

Interestingly, some studies investigated the impact of EOs on two or more risk factors for MetS rather than concentrating only on one risk factor. Cinnamon extracts (*Cinnamomum veru*) exhibited antidiabetic properties and had a beneficial impact on lipid profiles [82,83,84]. Hedge nettles from the Stachys species, commonly referred to as “mountain tea” are consumed in various parts of the world as a herbal tea to treat different medical conditions [85]. The bioactive compounds in this tea include germacrene D, β-pinene, α-pinene, hexahydrofarnesyl acetone, and valeranone. These compounds were observed to play a role in glucose homeostasis and prevent the absorption of fats by inhibiting enzymes such as cholinesterases, glucosidase, amylase, tyrosinase, and lipases respectively [86]. Similarly, lemon balm extract also prevented or concomitantly treated DM-associated dyslipidemia and hypercholesterolemia [87].

#### 3.2.5. Dosage, Bioactive Metabolites, Therapeutic, and Adverse Effects of EOs

We have reviewed the therapeutic effects, dosage, and possible side effects of EOs extracted from different sources such as peppermint, chamomile, fennel, rosemary, fenugreek, garlic, cumin, and lavender, among others, against various diseases, including microbial infections, obesity, diabetes, hypertension, and dyslipidemia (Table 1). Previous studies suggested that 225 mg/day of peppermint EOs may reduce the microbial dysbiosis and the total symptom score in patients with IBS [88,89,90]. Consumption of 100 mg/kg/b.w of chamomile EO prevents obesity and dyslipidemia, by reducing the weight gain and improve the lipid profile, and kidney and liver functions in animal models [91]. In addition, 3 g of chamomile EOs decreased the HOMA-IR index, serum HbA1C, TAG, TC, and LDL levels in patients with type 2 diabetes [92,93,94,95]. The use of 15 mg/kg/b.w and 7.5 mg/kg of fennel and rosemary EOs confer protection against hypertension and improve cardiac and renal function through anti-inflammatory and antioxidant activities [96,97]. Moreover, the consumption of 50 mg/kg/b.w of garlic EOs exerted anti-obesity and anti-hyperlipidemic effects by reducing HFD-induced body weight gain and adipose tissue weight [98,99]. The bioactive ingredients responsible for the therapeutic effects of EOs are polyphenols, flavonoids, tocopherols, menthone, tanins, luteolin, apigenin, transanethole, terpenoids, estragole, fenchone, limonene, diallyl disulfide, cuminaldehyde, α-pinene, γ-terpinene, linalool, and linalyl acetate [88,89,90,91,92,93,94,95,96,97,98,99,100,101,102]. The majority of these bioactive components have strong antioxidant activities, like phenolic compounds, terpenoids, luteolin, apigenin, estragole, fenchone, and limonene [91,92,93,94,95,101,102]. On the other hand, there are some side effects associated with the consumption of EOs. Therefore, it is important to know about the possible effects of EOs before using them. Previous studies demonstrated that EOs act as an endocrine disrupting chemical (EDC), an exogenous agent that interferes with hormonal release, action, storage, and metabolism in the body. However, EO may act as an agonist to the estrogen receptor alpha (ERα),antagonist to the androgen receptor (AR), and may cause endocrine disruption [103]. Regular exposure to lavender and tea tree oil is associated with abnormal breast growth and premature gynecomastia in adolescents [104,105]. Mild skin irritation was associated with consumption of chamomile tea in patients with type 2 DM [92].

### 3.3. Enhance Breast Milk Production and Childcare

Breastfeeding is beneficial for both mother and child from an immunological, physiological, psychological, and nutritional perspective [106,107]. However, poor milk production is considered one of the most common issues leading to early cessation of breastfeeding. Factors such as cesarean delivery, preterm birth, pain, fatigue, anxiety, return to work, emotional stress, and postpartum depression may affect milk production [108,109].

Medical and herbal galactagogues are often used to augment and increase milk production [110,111,112]. Nonetheless, aromatherapy accompanied by massage has been used historically as an alternative therapy [113,114]. Previous literature suggests that lavender oil has a comforting effect on the central nervous system, and a massage with lavender oil can increase milk production and prolactin levels in mothers [115,116,117]. A combination of aromatherapy and massage using EOs increased milk production to a larger extent, as compared to individual treatments [118]. Similarly, jasmine oil has been observed to increase milk secretion [119]. Furthermore, the application of menthol essence and peppermint is effective against nipple fissures common in lactating mothers [120,121].

EOs have also been observed to be beneficial during pregnancy and labor. Lemon EO (citrus lemon) effectively reduced nausea and vomiting during pregnancy [122]. Additionally, a randomized controlled trial observed that lavender aromatherapy massages helped reduce pain and the duration of labor [123]. Moreover, a triple-blind, randomized, placebo-controlled trial revealed that inhaling lavender essence may help decrease pain after a Caesarean section when used along with other medical and therapeutic approaches [124].

Literature reveals that EOs are also beneficial for infants and children. In one study, massaging with lavender oil was effective in relieving infants of the symptoms of colic [125]. During bathing, adding lavender oil reduced stress and crying and enhanced sleep in very young infants [126]. Using lavender aromatherapy during dental treatment decreased children’s anxiety and perception of pain [127]. Lavender oil was also effective in alleviating pain during blood sampling and vaccinations in children [128]. Similar results were shown in decreasing anxiety in children with diabetes using orange oil aromatherapy [129]. Aromatherapy using *Rosa damascena* EO showed improved sleeping quality in children with sleep disorders [130]. Moreover, EOs decreased chemotherapy-induced nausea and vomiting, decreased distress in burn patients, and the prevalence of rhinitis symptoms in children [131,132,133].

### 3.4. EOs: Natural Antibiotics

Foodborne pathogens are a common cause of foodborne illnesses that affect millions of people every year, sometimes with severe and lethal consequences [134]. As commonly used food preservatives are chemical in nature and may only be added up to a certain degree to prevent changes in taste/odor, there is a fair demand to identify naturally sourced compounds that would exert a similar effect without altering the organoleptic properties negatively [135]. Previously, extensive investigation into the use of EOs as food preservatives in the food industry has been conducted [52]. EOs have been observed to be effective against both pathogenic and non-pathogenic organisms [136,137,138,139,140,141]. They have been observed to be effective against gram-positive (*Staphylococcus aureus* and *Listeria monocytogenes*) and gram-negative bacteria (*Escherichia coli* and *Salmonella enteritidis*) [142]. Several studies conducted on food materials reported bactericidal or bacteriostatic activity of EOs against *Salmonella enterica, Escherichia coli* O157:H7, *Staphylococcus aureus*, *Listeria monocytogenes, Lactobacillus plantarum, Saccharomyces cerevisiae*, and *Candida albicans* strains [140,141,143,144]. Three citrus fruit EOs, mandarin (*Citrus reticulata*), wilking (*Citrus reticulata cv. Wilking blanco*), and clementine (*Citrus clementina*), were examined for their antimicrobial potential. Mandarin EO demonstrated the best bacterial growth inhibition, followed by clementine EOs [34]. The oils were significantly effective against *Candida albicans*, *Escherichia coli*, *Listeria* innocua, methicillin-resistant *S. aureus*, and *Staphylococcus aureus* [34]. Clove and melaleuca EO showed an inhibitory effect on S. *aureus*, *E. coli*, and C. *albicans* [145].

Phenolic EOs such as carvacrol, thymol, and others consist of hydrophobic ends that interact with different areas of microbial cells (e.g., cell wall and cytoplasmic membrane) and break the membrane structure, thereby causing a loss of cellular constituents and resulting in cell death [146].

### 3.5. Other Beneficial Effects of EOs

Mixtures containing the EOs of sage, oregano [147], *J. oxycedrus* subsp. *oxycedrus*, and *J. phoenicea* [148], have been reported to encourage wound healing. Similarly, a combination of sesame and lemon EOs accelerated the healing process of wounds in male Albino Wistar rats [149]. EOs have also been observed to cause subtle ST-segment elevation and decrease the levels of malondialdehyde and myeloperoxidase in rat models that had been induced with a myocardial infarction [150]. Malondialdehyde and myeloperoxidase are the markers that indicate necrosis of the myocardium; thus, the ability of EOs to reduce the levels of these enzymes points towards better heart health. Previous studies have also observed the positive effects of EOs on hepatic function. EOs of rosemary (*Rosmarinus officinalis* L.) and fennel (*Foeniculum vulgare*) have been associated with decreasing the levels of alanine aminotransferase (ALT) and alkaline phosphatase (ALP) [151,152]. These biomarkers indicate liver function insufficiency/damage. Acetominophen toxicity accounts for 50% of overdose-related acute liver failure and approximately 20% of liver transplant cases in the United States. EOs have also aided in treating such cases. EO extracted from *Nepeta cataria* L. increased the mRNA expression of uridine diphosphate glucuronosyltransferases and sulfotransferases [153]. These enzymes aid in metabolizing acetominophen into a nontoxic form that can be excreted through urine.

The beneficial effect of EOs on brain health has also been observed. Rosemary EO enhanced memory, concentration, alertness, and locomotor activity, besides stimulating the cerebral cortex and causing mood relaxation [154]. EOs from sage, rosemary, and Stachys inflata Benth inhibited acetylcholine esterase (AChE) even better than the drug donepezil [86,155]. This is a significant observation, especially for Alzheimer’s disease patients, as elevated activity of the enzyme (AChE) has been associated with the disease. The inhibitory potential of the AChE enzyme has been associated with the di- and triterpenes present in the EOs [155].

**Figure 3:**
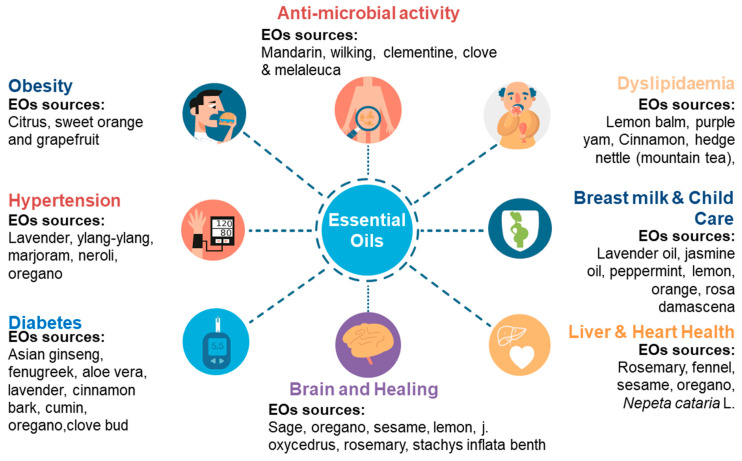
Sources of EOs and their respective health-promoting properties.

EOs have also been shown to exert beneficial effects on asthmatic patients; eucalyptol extracted from eucalyptus oil reduced the dependence on oral steroids by such patients [156]. Their usage has also been observed to reduce symptoms associated with primary dysmenorrhea [157] and labor pain [158]. Other benefits recorded include the ability to inhibit tyrosinase (anti-hyperpigmentation effect) [86], elastase (anti-wrinkle effect) [159], anti-viral [160], and anti-fungal activity [159,160]. Overall, EOs may positively change the course of many health issues, including obesity, dyslipidemia, hypertension, diabetes, and infections, along with improvements in brain, heart, and liver health (Figure 3).

## 4. Health-Associated Regulation and Consumption of EOs

Limited studies have examined the therapeutic efficacy, toxicity, and side effects of EOs. It is important to note that certain compounds in plants could be toxic, or can act as an irritant and cause allergic reactions [161]. The scarcity of human studies compared with the in vivo/in vitro studies makes it difficult to draw definite conclusions. There are many factors that jeopardize the use of EOs. These factors are apparent ambiguity of EOs, adverse effects and toxicity, challenges of identifying the active constituents causing the health benefits, their stability and the most important is the lack of scientific evidence supporting their use in humans [162]. Therefore, developing national and international policies and regulations on such products has become essential for both health authorities and the public. Such policies and regulations are needed to ensure the product’s safety, quality, efficacy, and reason for usage [163].

Although the legislative controls on EOs have not typically followed a structured model, both international and national bodies have laid down some basic rules regarding their usage. The prominent international organizations that are involved in the regulation of EOs include the World Health Organization (WHO), the Food and Agriculture Organization (FAO) of the United Nations, the FAO/WHO Codex Alimentarius Commission (CAC), and the International Organization for Standardization (ISO) [161]. The WHO works with international government and non-government stakeholders to develop strategies and implement regulations that ensure the safe use of EOs at the global level. The WHO undertook three main activities that can assist member states in fostering the safe use of complementary and alternative medicine (CAM):

1. WHO hosts a digital platform (WHO Essential Medicines and Health Products Information Portal) that addresses the safety of plant materials, including EOs. The portal includes 5480 medicines and health products and is updated every month.
2. The WHO regularly publishes guidelines regarding ‘good manufacturing product practices’ (GMP) for herbal medicines.
3. The WHO issued four volumes titled ‘The WHO monographs on selected medicinal plants to provide scientific information on the safety, efficacy, and quality of EOs and other natural products’.

The WHO and FAO established the Joint FAO/WHO Expert Committee on Food Additives (JECFA). The mandate of JECFA is to evaluate the safety levels of food additives, including EOs. This information is accessible to the general public and is available online using the committee’s website. On the other hand, FAO released a publication titled ‘Food and Agricultural Legislation’ (FAOLEX), which provides information on treaties, laws, and regulations on food, agriculture, and plant sources from all countries around the globe. Another joint effort by the WHO and FAO is the CAC, which is responsible for setting international standards, guidelines, and codes of food additives (keeping in perspective the health of the consumer) (http://www.codexalimentarius.org, accessed on 24 December 2020). The International Organization for Standardization (ISO) Technical Committee 54 addresses the standard specifications for EOs, mainly related to derivation, characterization, packaging, labeling, and storage. Further details about the roles and responsibilities of these international organizations are outlined in a previous review [161].

In addition to the international bodies that are involved in the regulation of EOs, national entities in various countries exist to assist in the proper regulation of these products (Table 2). Overall, within countries, the regulation of EOs depends mainly on their intended usage. The latter could be indicated by a claim on the product label, perceived by the consumer based on the reputation of this product, or deduced from the list of ingredients [164]. For instance, in the United States of America, the FDA reviews EOs under either cosmetic or drug categories depending on their use. Furthermore, in Japan, EOs indicated as fragrances or aromatherapy are imported as general merchandise and are not subject to any regulations. However, EOs used for cosmetics/drugs/quasi-drugs, or food flavoring are strictly controlled under national acts such as the Pharmaceutical Affairs Act and the Food Sanitation Act [165].

It remains important to note that besides the regulatory frameworks for the safe use of EOs, the global community has instilled a regulation aiming to protect endangered plant species under the Foreign Exchange and Foreign Trade Act, based on the Convention on International Trade in Endangered Species of Wild Fauna and Flora (CITES). The latter restricts or prohibits the importation of certain endangered plant species. For example, the EO of musk, which is obtained from the abdomen of musk deer, and the Brazilian rosewood oil are subject to this act.

Furthermore, researchers and the pharmaceutical industry focus their efforts on herbal products for economic reasons. As such, countries are directed toward legalizing the trade of these products across boundaries. However, many challenges may arise due to the lack of globally acceptable regulatory requirements for these products [178]. Therefore, global harmonization for the regulation of herbal products and their derivatives is needed to guide the responsible production and marketing of herbal medicines/ products. If sufficient scientific evidence of benefit is available for a herb, including extracts such as EOs, then legislation should allow for this to be used appropriately to promote public health and treat diseases [179].

## 5. Limitation and Future Direction

Although previous studies identified the beneficial health effects associated with the application of EOs, they have certain limitations. The majority of the studies haven’t reported the relative adverse effects or toxicity of EOs. The dosage and method of usage of EOs are not uniform. Very few randomized control trials exist for the evaluation of the efficacy and safety of these EOs in humans. In addition, the extraction methods for bioactive metabolites from plant EOs may significantly improve the therapeutic potential of EOs. However, these metabolites should be identified for their health benefits and safety. Furthermore, biological assays should clarify the effectiveness and validation of these compounds as antioxidants and anti-inflammatory agents. Since ancient times, EOs have been used in healing systems. The growth opportunity for the herbal product industry is on the rise across the globe due to consumer demand for herbal/natural products for healthcare, beauty, and diet supplementation.

## 6. Conclusions

In conclusion, preclinical studies have vastly documented the antioxidant, anti-inflammatory, anti-microbial, and anticancer activities of EOs, elucidating their mechanisms of action both at physiological and cellular levels. The source of the plant, dosage of EOs, and method of application may be altered to target a specific disease. Moreover, we have summarized that specific constituents present in EOs may exert these therapeutic effects in animal and human studies. However, more well-designed human clinical trials are needed to ascertain the actual efficacy and safety of EOs as pharmacological and nutraceutical agents.

## Figures and Tables

**Table 1:** Dosage, bioactive metabolites, therapeutic, and adverse effects of EOs.

Essential Oil	Experimental Condition	Usage/Dosage	Duration	Bioactive Compounds	Therapeutic Effects	Possible Side Effects/Toxicity	Reference
Peppermint	57 patients with IBS	Capsule/225 mg per day	4 weeks	Polyphenols, flavonoids, tocopherols, menthone, and tanins	Significant reduction in the total IBS symptoms score	Not detected	[88,89]
Chamomile	HFD-fed Wistar rats	Water extract/100 mg/kg b.w.	6 weeks	Phenolic compounds and terpenoids	Prevention of body weight gain; decrease in levels of serum TAG, TC, LPL, urea, and creatinine, ALT, and AST; decrease in MDA levels, increase in antioxidant enzyme activity (SOD, catalase, GPx) and GSH levels in the liver and kidney	Not detected	[90,91]
	64 patients with T2DM	Chamomile tea (3 g/150 mL hot water) three times per day	8 weeks	Luteolin and apigenin	Decrease in HOMA-IR index, serum HbA1C, insulin, TAG, TC, and LDL levels; no changes in HDL levels Decrease in serum MDA, increase in serum total antioxidant capacity and antioxidant enzyme activities (SOD, GPx, and catalase)	Mild adverse event	[92,93,94,95]
	HFD-fed C57BL/6J mice	10 mg/kg b.w.	12 weeks	Apigenin	Decrease in body weight, visceral fat weight, plasma lipid levels (TAG, TC, LDL), postprandial glucose levels, and reduction of hepatic SREBP-1c and SREBP-2 expressions	No side effect	[96]
Fennel and rosemary	48 male albino rats	Volatile oils-15 and 7.5 mg/kg b.w. respectively	4 weeks	Trans-anethole, terpenoids, estragole, fenchone, and limonene	Showed cardio and hepato- protective effect and safety towards kidney and blood sugar. Oxidative stress and inflammatory biomarkers were significantly improved	Not detected	[97]
Fenugreek	60 diabetic male Wistar rats	5% (*w*/*w*)	8 weeks	Terpenenes	Glucose, triglyceride (TG), and total-cholesterol (TC) and LDL-cholesterol (LDL-C) levels decreased significantly in the plasma and liver of diabetic rats and increased the HDL-Cholesterol (HDL-Ch) level, modulated key enzyme related to hypertension	Not detected	[98]
Garlic	HFD-fed C57BL/6J mice	50 mg/kg b.w.	12 weeks	Diallyl disulfide, DADS	Exerted anti-obesity and anti-hyperlipidemic effects by reducing HFD-induced body weight gain and adipose tissue weight	Not detected	[99]
Cumin	Male rats with hepatotoxicity	400 mg/kg	2 weeks	Cuminaldehyde, α-pinene and γ-terpinene	Normalized acetaminophen-induced liver enzyme elevation and preserved liver structure	Not detected	[100]
Lavender	75 diabetic neuropathic patients	Massage-2.5 cc of 3% lavender oil	4 weeks	Linalool and linalyl acetate	Significantly increase the quality of life domain, reduce neuropathic pain	Not detected	[101]
	52 diabetic patients with insomnia	Inhalation	4 weeks	Linalyl acetate and linalool	improve sleep quality and quantity, quality of life, and mood	Not detected	[102]

**Table 2:** List of agencies/organizations in selected countries that are involved in the regulation of EOs.

Country	Regulatory Agency
Australia	Therapeutic Goods Administration [166].
Canada	Food and Drugs Act (FDA) and the Natural Health Product Regulations (the Regulations) [167] by the Natural and Non-prescription Health Product Directorate (NNHPD).
China	However, herbal medicinal products are governed by the current Drug Administration Law to meet certain requirements before they are marketed [168,169].
European Union	The European Medicine Agency under directives 2001/83/EC and 2004/24/EC [170].
India	Drugs and Cosmetics Act (D and C) of 1940 and Rules of 1945, department of AYUSH [171].
Kingdom of Saudi Arabia (KSA)	The National Center for Complementary and Alternative Medicine (NCCAM) in Saudi Arabia, which is a part of the Ministry of Health (MOH) [172].
Philippines	The Food and Drug Administration (FDA) is the government agency that has regulatory power over the production, distribution, and use of herbal products [173].
South Africa	There are currently no guidelines or frameworks for the registration and regulation of traditional medicine (TM) and plant-based remedies in South Africa [174]. However, once a health-related claim is made for a finished product, it has to go through the full drug evaluation procedure at the Medicines Control Council (MCC) before marketing. Pharmaceutical standards need to be consistent with those of the United States Pharmacopoeia (USP) or the British Pharmacopoeia (BP) [175].
United States of America (USA)	The botanical products are classified as a drug, food, or dietary supplement by the United States Food and Drug Administration (FDA) based on the claims or end-use. As per the FDA, the drug must be marketed under an approved New Drug Application (NDA) [176]. The FDA regulates dietary supplements under the Dietary Supplement Health and Education Act of 1994. These do not require premarket approval, and it’s the responsibility of the marketer to ensure the safety and labeling compliance of their products with the regulations. The claims need to comply with the regulatory guidelines issued by the FDA. The manufacturing of dietary supplements should be performed as per the current Good Manufacturing Practices (GMP) for dietary supplements [177].

## Data Availability

Not applicable.
